# Structural Elucidation of the Reduced Mn(III)/Fe(III) Intermediate of the Radical-Initiating Metallocofactor in *Chlamydia trachomatis* Ribonucleotide Reductase

**DOI:** 10.1021/acs.biochem.4c00692

**Published:** 2025-02-17

**Authors:** Ryan J. Martinie, Jovan Livada, Nyaari Kothiya, J. Martin Bollinger, Carsten Krebs, Alexey Silakov

**Affiliations:** † Department of Chemistry, Hamilton College, Clinton, New York 13323, United States; ‡ Department of Chemistry, The Pennsylvania State University, University Park, Pennsylvania 16802, United States; § Department of Biochemistry and Molecular Biology, The Pennsylvania State University, University Park, Pennsylvania 16802, United States

## Abstract

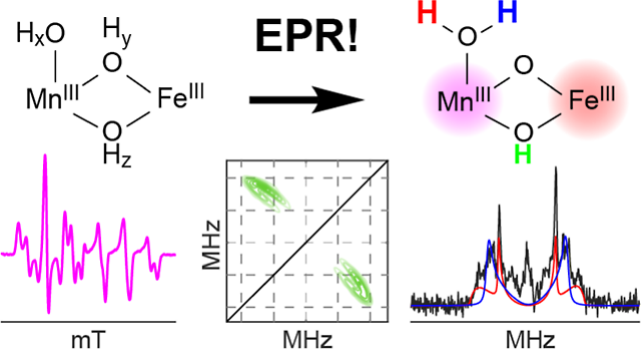

Ribonucleotide reductases (RNRs) are the sole *de novo* source of deoxyribonucleotides for DNA synthesis and repair across all organisms and carry out their reaction via a radical mechanism. RNR from *Chlamydia trachomatis* generates its turnover-initiating cysteinyl radical by long-range reduction of a Mn(IV)/Fe(III) cofactor, producing a Mn(III)/Fe(III) intermediate. Herein, we characterize the protonation states of the inorganic ligands in this reduced state using advanced pulse electron paramagnetic resonance (EPR) spectroscopy and ^2^H-isotope labeling. A strongly coupled deuteron is observed by hyperfine sublevel correlation (HYSCORE) spectroscopy experiments and indicates the presence of a bridging hydroxo ligand. Isotope-dependent EPR line broadening analysis and the magnitude of the estimated Mn–Fe exchange coupling constant together suggest a μ-oxo/μ-hydroxo core. Two distinct signals detected in electron–nuclear double resonance (ENDOR) spectra are attributable to less strongly coupled hydrons of a terminal water ligand to Mn(III). Together, these experiments imply that the reduced cofactor has a mixed μ-oxo/μ-hydroxo core with a terminal water ligand on Mn(III). This structural assignment sheds light generally on the reactivity of Mn/Fe heterobimetallic sites and, more specifically, on the proton-coupling in the electron transfer that initiates ribonucleotide reduction in this subclass of RNRs.

## Introduction

**Figure 1:**
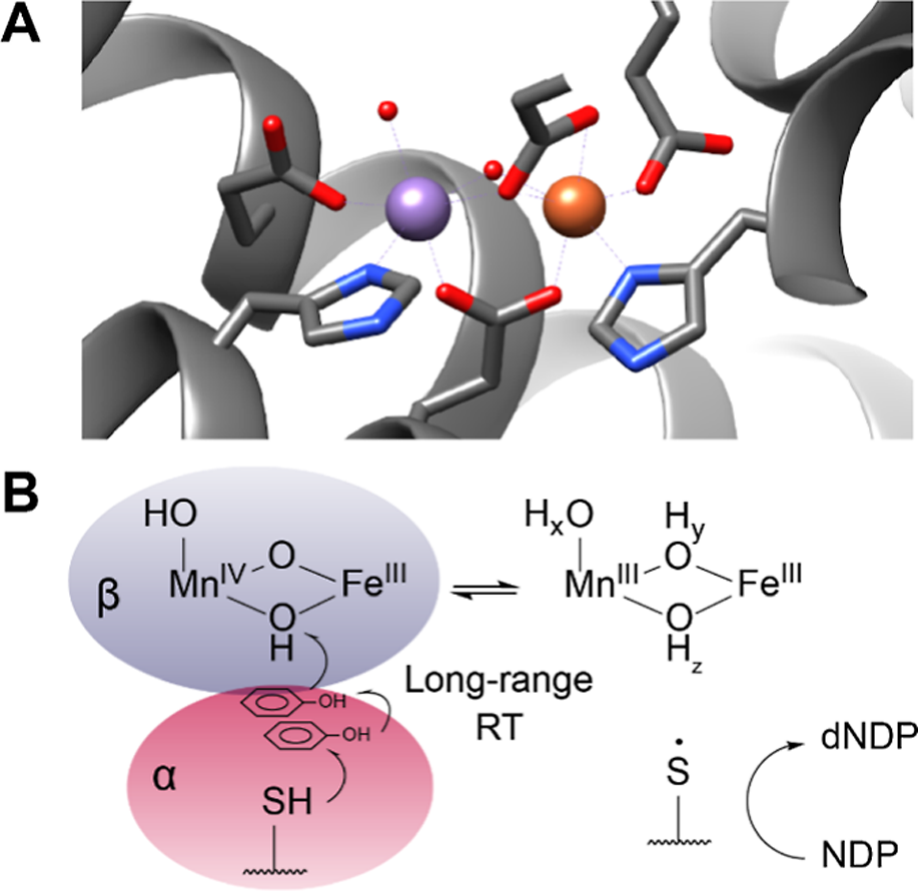
Active site (A) and catalysis (B) in the class Ic RNR from *C. trachomatis*. (A) Ribbon structure of the Mn(II)/Fe(II) state of *Ct* RNR β subunit, with coordinating residues depicted as sticks. PDB ID: 4M1I.^32^ Metal sites are termed site 1 (Mn, left) and site 2 (Fe, right). Atoms are color coded as Mn (magenta), Fe (rust), N (blue), O (red). Generated using UCSF Chimera.^33^ (B) Scheme of catalysis in *Ct* RNR. The Mn(IV)/Fe(III) cofactor in the β subunit (blue shading) oxidizes the cysteine in the α subunit (red shading) via a long-range radical translocation (RT), producing the Mn(III)/Fe(III) intermediate; the cysteinyl radical then initiates the conversion of a ribonucleotide (NDP) to the corresponding deoxyribonucleotide (dNDP, far right). Completion of the reaction reforms the cysteinyl radical, which oxidizes the Mn(III)/Fe(III) intermediate to produce the resting state. Oxygen ligands of unknown protonation state in the Mn(III)/Fe(III) intermediate are marked with the subscripts *x*, *y*, and *z*.

Ribonucleotide reductases (RNRs) convert ribonucleotides to 2′-deoxyribonucleotides. These enzymes constitute the sole *de novo* source of the deoxyribonucleotide precursors essential for DNA repair and replication in all organisms on earth.^1−3^ This reaction occurs via a radical mechanism initiated by the formation of a conserved cysteinyl radical, and RNRs are categorized by the various methods employed to generate this radical.^1,4,5^ In class I RNRs, present in humans and other eukaryotes and widespread in aerobic/facultative bacteria such as *Escherichia coli*, the cysteinyl radical is generated in the active site of the α subunit by electron transfer (ET) to a stable, one-electron oxidant in the β subunit of a heterotetrameric α_2_β_2_ active complex.^2,6−11^ Class II and III enzymes utilize an adenosylcobalamin cofactor and a glycyl radical cofactor, respectively, for radical generation.^7,12−16^ Class I RNRs are further subdivided by the identity of the one-electron oxidant in the β subunit.^17,18^ The canonical class Ia enzymes utilize a tyrosyl radical adjacent to a diiron site.^19−24^ Other members of class I utilize the dinuclear metal cluster as the oxidant, specifically a Mn/Fe heterobimetallic cofactor in class Ic and a dimanganese cofactor in class Id.^18,25,26^ The prototypical member of class Ic is the RNR from *Chlamydia trachomatis* (*Ct* RNR), which was initially identified by the presence of a Phe residue at the sequence position typically occupied by the tyrosyl radical oxidant of class Ia RNRs.^27^ Subsequent studies revealed that the active cofactor in *Ct* RNR is a Mn(IV)/Fe(III) heterobimetallic site (Figure 1),^25,28,29^ which can oxidize the cysteine residue in the α subunit to produce the reduced Mn(III)/Fe(III) form of the cofactor (Figure 1B).^25,30,31^ Upon completion of ribonucleotide reduction, the cysteinyl radical reforms, then oxidizes the Mn(III)/Fe(III) intermediate to regenerate the resting Mn(IV)/Fe(III) state.^30,31^

A major question in the field of bioinorganic chemistry is the manner in which enzymes are able to form potent reactive intermediates and carry out challenging chemistry while minimizing thermodynamically feasible but deleterious side reactions. Transfer of protons can profoundly alter the electrochemical potential of chemical species; given the ability of proteins to exclude water from interior active site pockets and to control proton access via gated hydrogen-bonding networks and conformational changes, protonation state is often invoked as a mechanism for enzyme control of reactive intermediates. Yet, the experimental determination of protonation state, particularly for a reactive species in a biological system, is generally nontrivial, and the history of metallo-enzymology is replete with examples of controversy over the precise structural assignment of intermediates of interest.^34−43^

Intermediates in the catalysis of class I RNRs are among the species that have attracted attention. In the class Ia RNR from *E. coli*, the active diferric tyrosyl radical cofactor is formed by reaction of dioxygen with the diferrous form of the enzyme via a reactive Fe(IV)/Fe(III) intermediate termed “*X*”.^44−46^ The structure of *X* has attracted considerable interest spanning decades, and it ultimately accepts an electron from the adjacent tyrosine to form a tyrosyl radical and a diferric form of the enzyme.^40−43,46−50^ Once formed, catalysis is initiated when the tyrosyl radical oxidizes the cysteine in α via a bidirectional, long-range radical translocation (RT).^8,9^ This intersubunit RT occurs across ∼35 Å,^10,11,51−54^ though enzyme turnover is too fast to be accounted for by a single tunneling ET step of this distance.^55^ Instead, the RT proceeds via a series of transient “stepping stone” radicals at conserved redox-active residues spanning both subunits, with shorter ET steps between each. Although direct detection of these intermediate radicals is precluded by a rate-limiting conformational change, pathway radicals have been detected by incorporation of unnatural amino acid analogs with altered redox properties and p*K*_a_s.^9,56−60^ It has been proposed that each of the constituent ET steps is coupled to proton transfer and that this coupling may allow the enzyme to tune the thermodynamics of the overall RT process to ensure efficiency and bi-directionality.^61−64^ The first proton transfer step was examined by characterization of the post-RT state using Mössbauer spectroscopy; this study showed that the diiron cofactor is involved not only in the generation of the tyrosyl radical, but also as a proton donor to facilitate the long-range RT in a proton-coupled manner in each catalytic cycle.^24^

Compared to the class Ia enzyme from *E. coli*, less is known regarding proton control over reactive intermediates and RT in *Ct* RNR. The active Mn(IV)/Fe(III) state consists of high-spin Mn(IV) and Fe(III) ions antiferromagnetically coupled to yield an overall spin of *S* = 1;^28^ the Mn(IV) ion is present in “site 1” of the dimetal cofactor and therefore possesses an open coordination site occupied by a nonprotein oxygen (i.e., oxo/hydroxo/water) ligand (Figure 1).^65^ This species is formed when the Mn(II)/Fe(II) state of the enzyme reacts with O_2_ to yield a Mn(IV)/Fe(IV) intermediate with spin *S* = 1/2.^66,67^ Through the complementary application of X-ray absorption spectroscopy (XAS), X-ray emission spectroscopy (XES), and advanced pulse electron paramagnetic resonance (EPR) spectro-scopy, the Mn(IV)/Fe(IV) state was shown to possess a di-μ-oxo “diamond core” with a terminal hydroxo ligand to Mn(IV).^68,69^ ET from an external electron donor converts this intermediate to the active Mn(IV)/Fe(III) cofactor.^30,67^ Extended X-ray absorption fine structure (EXAFS) analysis was used to show that, upon conversion of the cofactor to the Mn(IV)/Fe(III) state, the Mn–Fe distance is 2.92 Å [increased from 2.78 Å in Mn(IV)/Fe(IV)^68^], most consistent with a μ-oxo/μ-hydroxo core (Figure 1B).^70^ These EXAFS measurements did not shed light on the protonation state of the terminal ligand to Mn(IV), though in a later study magnetic circular dichroism (MCD) and nuclear resonance vibrational spectro-scopy (NRVS) data were interpreted as most consistent with a μ-oxo/μ-hydroxo core and a terminal hydroxo ligand;^71^ valence-to-core XES measurements were also found to be consistent with this model.^69^ By contrast, little has been reported regarding the RT portion of the *Ct* RNR reaction. The distance of the RT has been measured directly by forming the product of the RT reaction with the irreversible, mechanism-based inactivator N_3_UDP (which produces a stable nitrogen-centered radical, N^•^, in the active site of α) and performing double electron–electron resonance (DEER) measurements between the *S* = 1/2 Mn(III)/Fe(III) species in β and the N^•^ inactivation product.^72,73^ This distance was measured to be 43 Å, consistent with an α_2_β_2_ heterotetrameric docking model analogous to that initially proposed for *E. coli* class Ia RNR.^73^

The RNR from *C. trachomatis* was the first biological system to be shown to employ a Mn/Fe redox cofactor; however, in recent years, a number of additional Mn/Fe-dependent proteins have been discovered. These include: R2lox, a protein of unknown *in vivo* function that forms a post-translational Tyr–Val cross-link;^74,75^ AibH2, which catalytically mono-oxygenates 2-aminoisobutyric acid;^76^ and SfbO, which was shown to carry out monooxygenase chemistry on a substrate surrogate.^77^ Of these, R2lox is the most well-characterized.^74,75,78−83^ The resting state of R2lox consists of an antiferromagnetically coupled Mn(III)/Fe(III) site with two carboxylate (one from the protein and a second from a bound fatty acid) and one hydroxo bridging ligands.^74,78^ Intermediates in the formation of this resting state and the installation of the cross-link have also been evaluated; transient optical absorption and continuous-wave (CW) EPR features suggest that oxygen addition forms a μ-peroxo-Mn(III)/Fe(III) species (*I*_1_) which converts to a high-valent species responsible for activation of the adjacent Tyr/Val (*I*_2_).^80^ Cross-link formation is accompanied by formation of a μ-oxo/μ-hydroxo-Mn(III)/Fe(III) species (*I*_3_); this species then forms a μ-hydroxo-Mn(III)/Fe(III) intermediate (*I*_4_), and binding of a fatty acid to *I*_4_ yields the resting state.^83^ Both AibH2 and SfbO were shown to assemble a Mn/Fe heterobimetallic site and produce a Mn(III)/Fe(III) species upon exposure to oxygen.^76,77^ This expanding backdrop of Mn/Fe biochemistry raises new questions regarding the scope of chemistry available to this chemical manifold and the manner in which biology is able to control and tune the reactivity of these sites. *Ct* RNR, as the founding member of this cadre of proteins, provides an important point of comparison for further exploration in this emerging area.

A combination of crystallographic and spectroscopic experiments have provided key insights into the structure of the Mn(II)/Fe(II), Mn(IV)/Fe(IV), and Mn(IV)/Fe(III) states of *Ct* RNR, but conspicuous by its absence is structural information regarding the Mn(III)/Fe(III) state, one of the two states of the cofactor [together with Mn(IV)/Fe(III)] that are directly involved in bidirectional RT and, thereby, the catalytic production of deoxyribonucleotides. In this work, we furnish such structural insight by characterizing the protonation states of the inorganic ligands in the Mn(III)/Fe(III) form of *Ct* RNR by advanced pulse EPR spectroscopy. Using a combination of hyperfine sublevel correlation (HYSCORE) spectroscopy, electron–nuclear double resonance (ENDOR), and CW EPR line broadening and power-saturation experiments, we assign the protonation states of the various oxygen ligands in the Mn(III)/Fe(III) state. This structural assessment further illuminates the structure–reactivity relationship in the Mn/Fe heterobimetallic manifold and illustrates the role of protonation in long-range radical translocation and deoxyribonucleotide production in *C. trachomatis*.

## Results and Discussion

**Figure 2:**
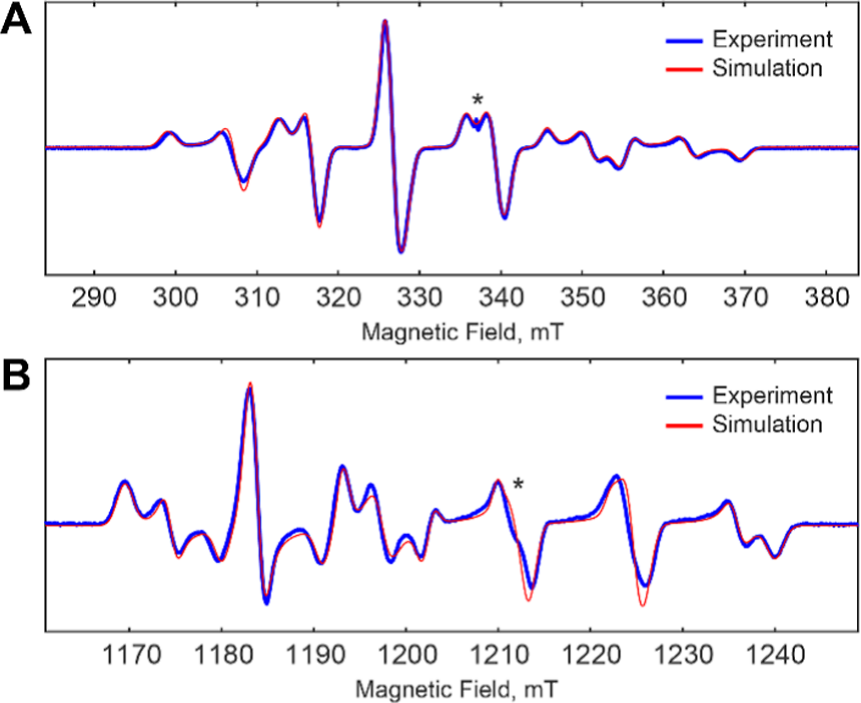
Electron paramagnetic resonance spectra of the Mn(III)/Fe(III) state of *Ct* RNR. (A) X-band continuous-wave EPR spectrum obtained at a microwave frequency of 9.435 GHz, microwave power of 100 μW, modulation amplitude 0.15 mT, and a temperature of 15 K. (B) Derivative of a pulse EPR spectrum obtained by monitoring the integral of free-induction-decay after a 1 μs microwave pulse. This spectrum was collected at a microwave frequency of 33.991 GHz and a temperature of 14 K. In both panels, experimental spectra are shown in blue with simulations in red; simulations used the following parameters: *g* = [2.0264, 2.0157, 2.0103] and *A*_55Mn_ = [269.7, 398.2, 314.4] MHz. A minor organic radical contaminant is denoted by an asterisk at *g* ∼2.

The Mn(III)/Fe(III) form of *Ct* RNR does not significantly accumulate under catalytic conditions, but it can be generated in one of the two αβ pairs of the tetramer by inducing RT and trapping the radical before it returns to the Mn/Fe site, either in the active site of α using N_3_UDP or by intercepting it within the RT pathway using hydroxyurea.^25,72,84^ Because both of these methods leverage the native RT machinery of RNR to generate the Mn(III)/Fe(III) state and produce indistinguishable, well-defined EPR signals,^25,84^ it is generally presumed that the resulting species is identical to the native Mn(III)/Fe(III) RT product. In the present study, the Mn(III)/Fe(III) state was obtained by incubation of the α and β subunits with substrate in the presence of hydroxyurea, as previously described, because this method produces samples that lack the contaminating EPR signal from the N^•^ produced by N_3_UDP treatment.^84^ EPR spectra for the resulting Mn(III)/Fe(III) samples were collected at X- and Q-band (Figure 2A and B, respectively, blue spectra). As previously reported,^25,73^ the Mn(III)/Fe(III) state exhibits a complex six-line EPR spectrum arising from strong hyperfine coupling to ^55^Mn (*I* = 5/2). The multifrequency spectra can be globally simulated with *g* = [2.0264, 2.0157, 2.0103] ± 0.0001 and *A*_55Mn_ = [269.7, 398.2, 314.4] ± 2 MHz (Figure 2, red spectra), in reasonable agreement with previous reports.^25,73^ Notably, the ability to simulate both spectra with a single set of parameters with respect to the *S* = 1/2 ground state suggests that the exchange coupling between the two metals is large and the *S* = 1/2 ground state is well isolated; we experimentally probe this coupling and discuss its implications below.

**Figure 3:**
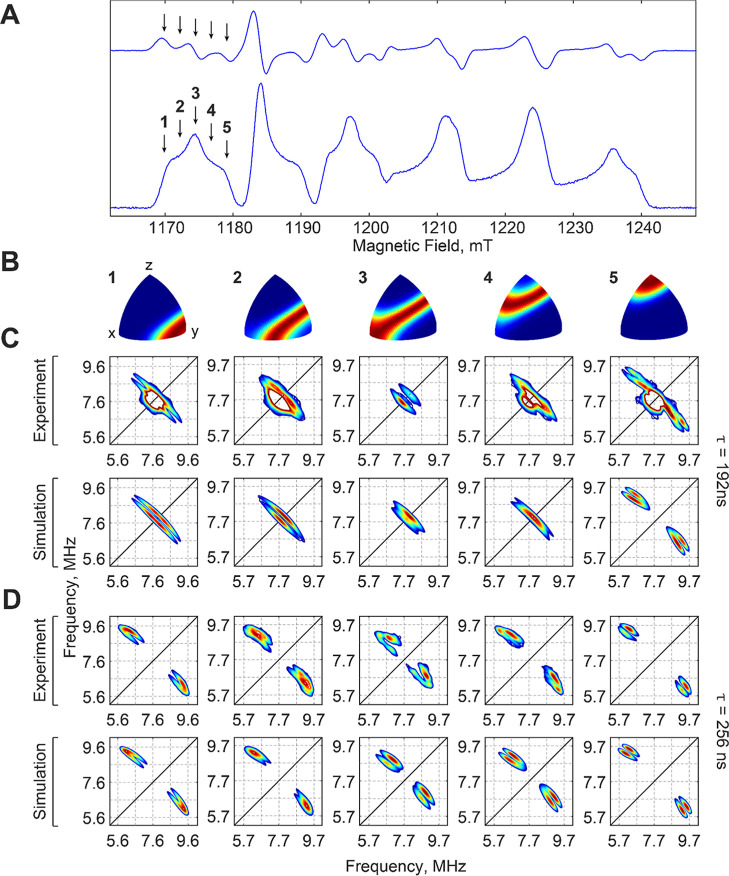
Orientation-selective, Q-band ^2^H-HYSCORE characterization of the Mn(III)/Fe(III) state of *Ct* RNR prepared in ^2^H_2_O. (A) One-dimensional EPR spectrum indicating the magnetic field positions (arrows, 1–5) at which HYSCORE spectra were acquired. Spectrum is depicted in both derivative (above) and absorption (below) formats for clarity. (B) Calculated orientation selectivity maps (1–5) resulting from HYSCORE acquisition at the magnetic field positions shown in (A). Color coding: red, greatest excitation; blue, no excitation. (C) Orientation-selective ^2^H-HYSCORE spectra (top row) and corresponding simulations (bottom row) acquired at (from left to right): 1169.9 (1), 1172.0 (2), 1174.2 (3), 1176.7 (4), and 1179.1 (5) mT, microwave frequency 33.99 GHz, 14 K, and τ = 192 ns. Contour levels are set to correspond to the maxima of the strongly coupled signals, with minima set at 40% of the corresponding maximum. (D) Orientation-selective ^2^H-HYSCORE spectra (top row) and corresponding simulations (bottom row) acquired at (from left to right): 1169.8 (1), 1172.1 (2), 1174.5 (3), 1176.7 (4), and 1179.5 (5) mT, microwave frequency 33.99 GHz, 14 K, and τ = 256 ns. Minimum contour levels are set at 40% of maximum.

To examine the protonation of the oxygen ligands in the Mn(III)/Fe(III) state, the protein was exchanged into ^2^H_2_O prior to EPR sample preparation to differentiate hydrons at solvent exchangeable positions (^2^H in this sample) and nonexchangeable positions (which remain predominantly ^1^H). Orientation-selective HYSCORE spectra reveal the presence of anisotropic hyperfine couplings to multiple exchangeable hydrons (Figure 3). Measuring HYSCORE spectra on the low-field portion of the EPR spectrum (Figure 3A) allowed us to obtain a complete set of orientation-selective data (Figure 3B). The spectra collected with a delay time τ = 192 ns (Figure 3C) reveal the presence of a relatively strongly coupled signal centered on the ^2^H Larmor frequency (∼7.7 MHz at these magnetic fields), with overall width of ∼2–4 MHz along the antidiagonal (Figure S1). As expected, splitting along the diagonal is also observed due to quadrupolar coupling to the *I* = 1 ^2^H nucleus. Signals from more weakly coupled ^2^H nuclei are also resolved (Figure S2) and are discussed in more detail below. To ensure that the outer reaches of the strong coupling were not obscured by the τ-dependent blindspot effect (blindspots are present at ∼5.2 and 10.4 MHz when τ = 192 ns), equivalent HYSCORE spectra with τ = 256 ns (blindspots expected at ∼3.9, 7.8, and 11.7 MHz) were also recorded. These latter conditions suppress the weak couplings and more effectively reveal the breadth of the larger coupling (Figure 3D). The parameters of the strongest ^2^H hyperfine coupling in the HYSCORE spectra can be extracted by global simulation of all obtained spectra (Figure 3C,D), yielding hyperfine coupling *A*_2H_ = [3, −4, 4] MHz with Euler angles [45, 5, 0]° and quadrupole coupling *Q*_2H_ = [0.056, 0.084, −0.140] MHz with Euler angles [−45, −100, 0]° (Table 1, Hydron 1).

**Table 1:** Parameters of Reported Hyperfine Coupling Simulations^a^

hydron	*A* (MHz)			Euler (deg)			*Q*		Euler (deg)		
	*x*	*y*	*z*	ϕ	θ	ψ	*K* (MHz)	η	ϕ	θ	ψ
1 (^2^H)	3 ± 1 [20]	–4 ± 0.3 [−26]	4 ± 0.3 [26]	45 ± 10	–5 ± 10	0 ± 10	0.07 ± 0.01	0.2 ± 0.2	–45 ± 20	–100 ± 10	0 ± 10
2 (^1^H)	4.2 ± 0.2	4 ± 0.2	–8 ± 0.3	0 ± 40	55 ± 10	45 ± 15					
3 (^1^H)	4 ± 0.2	6 ± 0.2	–6.5 ± 0.5	0 ± 10	85 ± 20	80 ± 20					

a Error margins estimated based on deviation that produces an unacceptable simulation. For ^2^H coupling (hydron 1), equivalent ^1^H coupling magnitudes are indicated in square brackets. Quadrupolar coupling parameters are reported according to *Q*_2H_ = [1 – η, 1 + η, −2] × *K*.

The magnitude of this coupling suggests that it arises from one or more exchangeable hydrons very close to the Mn/Fe cluster. Given that the Mn(IV)/Fe(III) state is known to possess a bridging hydroxo ligand,^69−71^ the presence of an analogous moiety in Mn(III)/Fe(III) is highly plausible. The dipolar coupling of a bridging hydroxo hydron to metal centers with *S* = 5/2 and *S* = 2 that couple anti-ferromagnetically to yield overall spin *S*_tot_ = 1/2 has been evaluated extensively for the case of intermediate *X* in the class Ia RNR from *E. coli*.^43,47^ In this model, the overall hyperfine coupling is expected to be highly anisotropic, with a maximum hyperfine coupling constant of ∼4 MHz for ^2^H (∼26 MHz for ^1^H) and mostly rhombic character. Considering the uncertainty margins (Table 1), the hyperfine coupling extracted from our HYSCORE experiments conforms well with this model, albeit with lower rhombicity than predicted. However, we note that this model neglects the presence of spin density on the oxygen atom of the hydroxo ligand,^85^ which could substantially modify the character of the hyperfine coupling. Moreover, couplings of similar magnitude (2.5–4.5 MHz) have been observed in Q-band ^2^H-HYSCORE spectra of R2lox in various Mn(III)/Fe(III) states directly comparable to that of *Ct* RNR and assigned as arising from a μ-hydroxo hydron.^78,81,83^ These HYSCORE experiments therefore indicate the presence of a μ-hydroxo ligand; however, this analysis alone cannot establish whether this signal arises from a single bridging hydroxo (e.g., from a μ-oxo/μ-hydroxo core) or from two hydrons with nearly identical coupling parameters (presumably arising from a di-μ-hydroxo core).

To address whether the strongly coupled signal in the HYSCORE experiments arises from one or two hydrons, we evaluated the change in line-broadening in X-band CW EPR experiments resulting from preparation of the sample in ^2^H_2_O as opposed to natural abundance solvent. Similar approaches have previously been reported using ^17^O/^16^O, rather than ^2^H/^1^H.^85,86^ As expected, the two samples give highly similar spectra, but modest broadening is apparent in the spectra of the H_2_O sample relative to that of the ^2^H_2_O sample (Figure S3); the unusual ^2^H_2_O-induced broadening previously seen in R2lox and some related model complexes is not present.^83,87^ Quantitative EPR line shape analysis (Figure S4 and Table S1) is completely compatible with the presence of one, but not two, ^2^H coupling with the parameters extracted from the HYSCORE spectra (for additional discussion of the fitting method and parameters, see Supporting Information). The HYSCORE signals analyzed above therefore arise from a single μ-hydroxo ligand.

**Figure 4:**
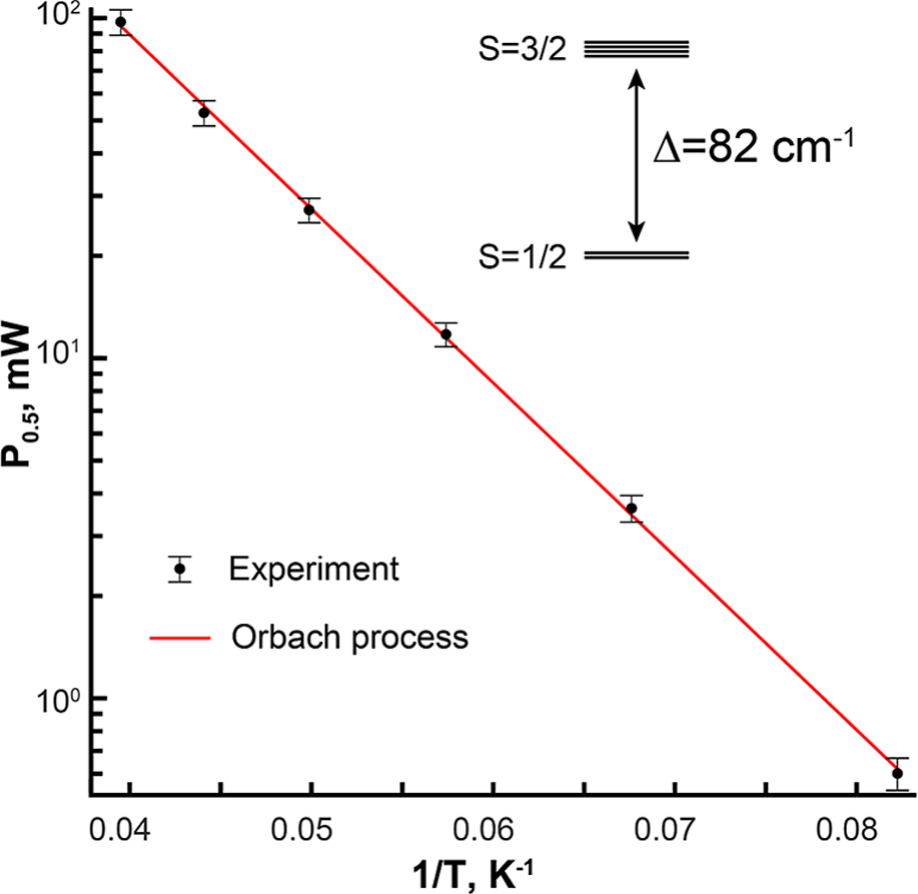
Temperature-dependence of CW EPR power saturation behavior for the Mn(III)/Fe(III) form of *Ct* RNR.

We also performed temperature-dependent power-saturation experiments in X-band CW EPR (Figure 4). This analysis aimed to resolve the magnitude of the spin–spin interaction (*J*) between Mn(III) and Fe(III), which is highly dependent on the nature of the bridging ligands. Strong antiferromagnetic spin–spin interaction in dimetal systems with oxygen ligands (|*J*| > 40 cm^–1^, *Ĥ = J***S**_**1**_**·S**_**2**_ convention used throughout) is typically facilitated by a μ-oxo ligand, whereas compounds bridged solely by hydroxo and water ligands typically exhibit a substantially weaker spin–spin interaction (|*J*| < 20 cm^–1^). For instance, in the analogous R2lox system, two Mn(III)/Fe(III) states have been characterized as lacking an oxo bridge (the wild-type resting state and *I*_4_); these states exhibit exchange couplings of ∼9 and ∼5 cm^–1^, respectively.^78,81,83,88^ In contrast, two additional Mn(III)/Fe(III) states in R2lox have been assigned as possessing a μ-oxo ligand (*I*_3_ and the resting state of the G68L variant) and have larger exchange couplings of 80 and 33 cm^–1^, respectively.^81,83^ In the present case, the *P*_0.5_(*T*^–1^) dependence plotted on a log_10_(*P*_0.5_) scale (Figure 4) shows a linear dependence indicative of an Orbach relaxation process that depends on the energy of the first excited spin state (*S* = 3/2) relative to the *S* = 1/2 ground state. Fits to this linear relationship yielded a Δ = 82 ± 4 cm^–1^, corresponding to a value of |*J*| = 54 ± 3 cm^–1^ (for additional details regarding the fitting process and analysis, see Supporting Information). An exchange interaction of 50–60 cm^–1^ suggests the presence of at least one oxo bridge, ruling out models with a dimetal core bridged solely by hydroxo, water, and carboxylate ligands. This finding provides an independent experimental basis for disfavoring a di-μ-hydroxo core and weighs against structural proposals with a single oxo/hydroxo bridge (which would have to be a hydroxo on the basis of HYSCORE experiments, vide supra). Thus, the estimation of the exchange coupling constant in the Mn(III)/Fe(III) state, together with the line-broadening analysis presented above, is most consistent with an assignment of a mixed μ-hydroxo/μ-oxo core.

**Figure 5:**
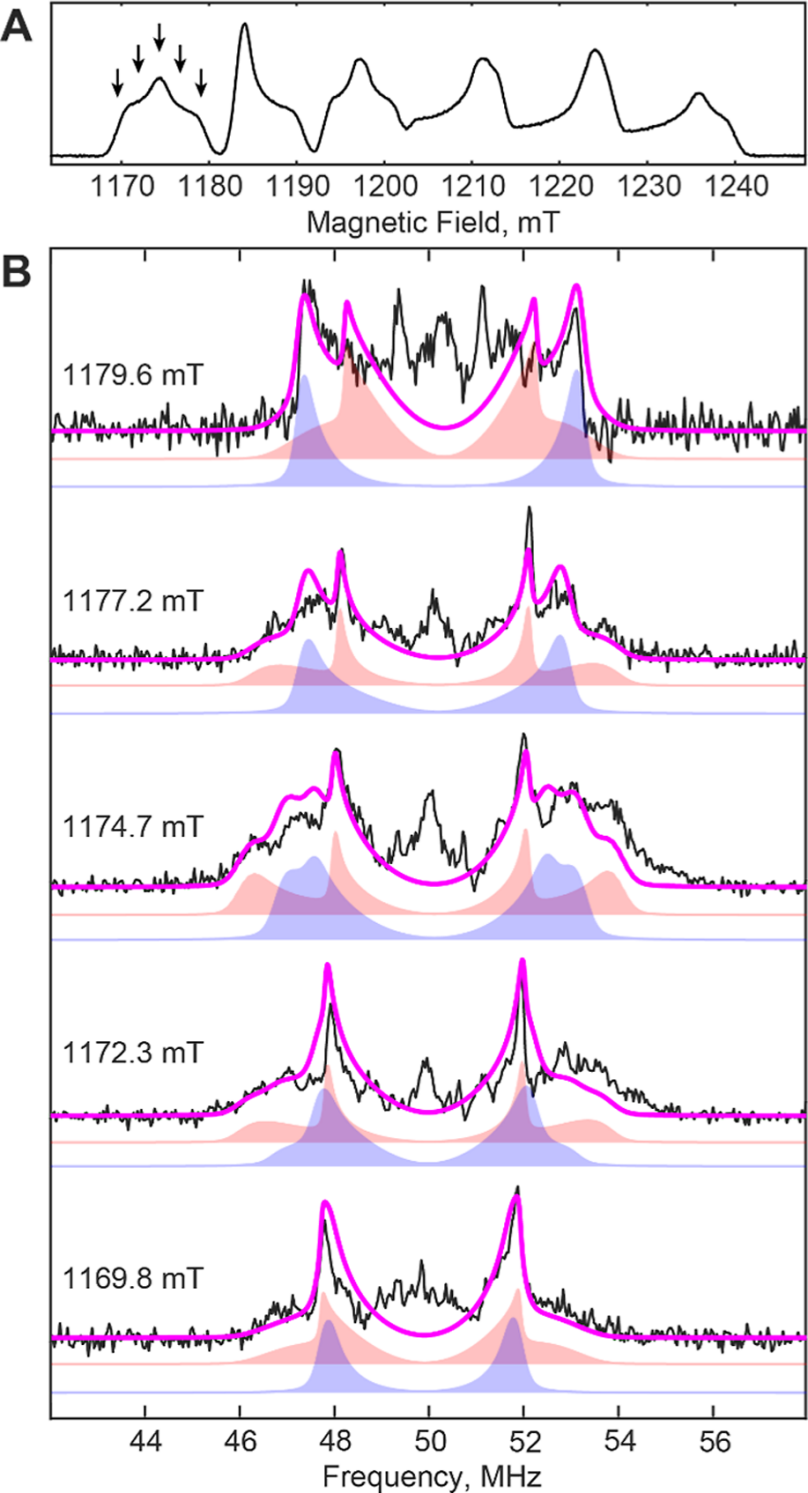
Q-band, ^1^H-ENDOR spectroscopic characterization of the Mn(III)/Fe(III) state of *Ct* RNR. (A) One-dimensional EPR spectrum indicating the magnetic field positions (arrows) at which ENDOR spectra were acquired. Spectrum was acquired by monitoring the intensity of a free-induction-decay (FID) as a function of the magnetic field. (B) Orientation-selective Mims ENDOR spectra (black) and simulation (magenta). Experimental data were acquired by subtracting the spectrum of a sample prepared in ^2^H_2_O from that of a sample prepared in natural abundance solvent, measured under identical conditions. Simulation parameters are given in Table 1; the magenta trace (simulation) represents the sum of the contributions from two overlapping signals, which are shown offset (hydron 2, red shading; hydron 3, blue shading). Central features (∼50 MHz) arise from weak hyperfine couplings attributable to matrix protons. Spectra were collected using the refocused Mims method with a microwave frequency of 33.991 GHz, τ = 76 ns, *t*_π,RF_ = 12 μs and a temperature of 14 K.

The above experiments strongly suggest that the Mn(III) and Fe(III) ions are bridged by an oxo and a hydroxo ligand; we also sought to characterize the protonation state of the terminal ligand to Mn(III) (Figure 1). As mentioned above, comparatively weak couplings are clearly present in the ^2^H-HYSCORE spectra (Figure S2), but the overlap with the stronger coupling (as well as those from the inevitable matrix ^2^H nuclei) complicates analysis of these signals. To probe these weaker couplings more robustly, we obtained orientation-selective ^1^H-ENDOR spectra using the refocused Mims method for Mn(III)/Fe(III) samples prepared in natural abundance solvent and those exchanged into ^2^H_2_O.^89^ By subtracting the spectra from the sample prepared in ^2^H_2_O from that in ^1^H_2_O (Figure S5), we obtained ^1^H-ENDOR spectra specific to protons at exchangeable positions (Figure 5).

The resulting experimental spectra could not be satisfactorily simulated with a single set of hyperfine parameters (Figure S6); for additional details see Supporting Information. However, introduction of a second set of approximately axial parameters yielded a fit that reproduces the key features of the experimental spectra (Figure 5). This simulation utilizes the following parameters: *A*_1H,2_ = [4.2, 4.0, −8.0] MHz with Euler angles [0, 55, 45]° and *A*_1H,3_ = [4.0, 6.0, −6.5] MHz with Euler angles [0, 85, 80]° (Table 1). It should be noted that these are ^1^H hyperfine couplings, which would correspond to ^2^H couplings of *A*_2H,2_ = [0.9, 0.9, −1.8] MHz and *A*_2H,3_ = [0.9, 1.3, −1.4] MHz, in good agreement with the overall width of the weak coupling(s) observed in the ^2^H-HYSCORE spectra (widths of ∼0.7–1.4 MHz, Figure S2). The observed couplings are too small to arise from the proton of a bridging hydroxo ligand (*vide supra*) and too large for exchangeable hydrons on amino acid residues in the first- and second-coordination spheres (the closest of which are expected to be >5.0 Å away, giving rise to couplings *A*_1H_ <1 MHz).^68,90^ In contrast, the observed couplings conform to expectations for the protons on a terminal hydroxo/water ligand to Mn(III);^90^ the presence of two distinct couplings allows assignment of this terminal ligand as water. The observed couplings to a terminal water ligand are smaller than those assigned as arising from a terminal water ligand in the resting state of R2lox. We attribute this apparent discrepancy to differences in the electronic structure, as corroborated by the different *g*-anisotropy, Mn hyperfine, and *J* coupling.

**Figure 6:**
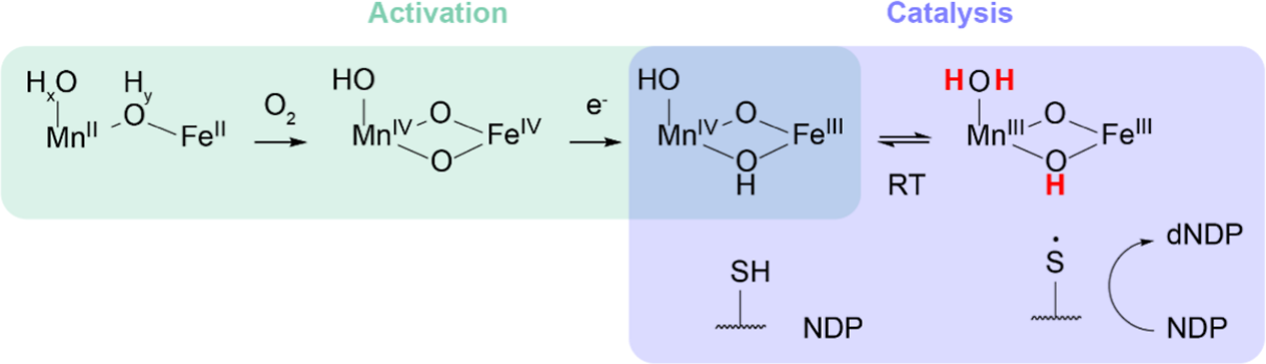
Model for the protonation states of the metallocofactor in activation and catalysis of *Ct* RNR, based on currently available data. The activation reaction is highlighted with green shading, whereas the catalytic reaction is highlighted with blue shading. Protonation states for the Mn(III)/Fe(III) state determined in this study are shown in bold red. Unassigned protonation states in the Mn(II)/Fe(II) state are indicated by subscripts *x* and *y*.

Together, the above experiments allow the complete assignment of the protonation state of the Mn(III)/Fe(III) state of *Ct* RNR: a μ-oxo/μ-hydroxo core with a terminal water ligand to Mn(III) (Figure 6). This assignment aligns with current knowledge of the other known states in *Ct* RNR catalysis. The Mn(IV)/Fe(IV) activation intermediate has a di-μ-oxo core with a terminal hydroxo ligand to Mn(IV).^68^ A variety of spectroscopic experiments indicate that the Mn(IV)/Fe(III) cofactor contains a μ-oxo/μ-hydroxo core with a terminal hydroxo, corresponding to the addition of a single proton to one of the bridging oxo ligands upon reduction of the Mn(IV)/Fe(IV) intermediate (Figure 6).^69−71^ Reduction of the Mn(IV)/Fe(III) cofactor to produce Mn(III)/Fe(III) in the course of catalysis would reasonably be accompanied by addition of one proton, and indeed the results reported herein suggest the conversion of the terminal hydroxo of Mn(IV)/Fe(III) to a water ligand in Mn(III)/Fe(III) (Figure 6). The source of this proton is a matter of speculation, as a proximal donor is not immediately apparent upon examination of the structure. Cofactor ligands E227 and E89 are both likely to form hydrogen bonds to the hydroxide/water ligand to the Mn ion,^68,90^ though neither is expected to be appreciably protonated under physiological conditions, and thus presumably could act as a relay from another source (such as the water molecule hydrogen bonded to ligand E193).^32^ Regardless of the precise source of the proton, it is notable that the proton transfer observed here is the inverse of that observed in *E. coli* RNR. In both cases, reduction of the stable oxidized cofactor in β is accompanied by its protonation; in *E. coli* β the terminal water ligand to the Fe(III)/Fe(III) cluster functions as a proton donor to the tyrosyl radical, whereas in *C. trachomatis* β, the terminal hydroxo ligand to the Mn(III)/Fe(III) cofactor is the proton acceptor.^24^

With considerable characterization of the Mn(II)/Fe(II),^32^ Mn(IV)/Fe(IV),^66,68,69^ and Mn(IV)/Fe(III) states having previously been reported,^25,28,69−71^ the structural elucidation of the Mn(III)/Fe(III) state fills a prominent gap in our understanding of *Ct* RNR catalysis. This insight is particularly relevant since the Mn(III)/Fe(III) state is [together with Mn(IV)/Fe(III)] one of the two states involved in the catalytically essential, bidirectional RT step (Figure 6). The observation of an additional proton in the Mn(III)/Fe(III) state relative to the Mn(IV)/Fe(III) resting state also constitutes, to the authors’ knowledge, the first experimental evidence of proton coupling to the RT step in *Ct* RNR, as was previously shown for the *E. coli* enzyme. Moreover, the structural characterization of the full suite of known oxidation states of this heterodinuclear cofactor provides a useful framework for the exploration of analogous intermediates in other Mn/Fe enzymes with varied reactivity as they continue to be discovered, serving as a foundation for the understanding of structure–function relationships in this chemical manifold.

## Conclusions

Advanced pulse electron paramagnetic resonance methods have been utilized to characterize the interactions between the *S* = 1/2 Mn(III)/Fe(III) state of *C. trachomatis* RNR and nearby exchangeable hydrons. ^2^H-HYSCORE measurements at Q-band reveal the presence of a strong hyperfine coupling interaction consistent with at least one bridging hydroxo ligand; CW EPR line broadening suggests this HYSCORE signal arises from a single μ-hydroxo ligand. Temperature-dependent relaxation experiments indicate the presence of a μ-oxo ligand, allowing the unambiguous assignment of a mixed μ-oxo/μ-hydroxo core. ^1^H-ENDOR measurements reveal the presence of two weaker hyperfine interactions, of which the most chemically reasonable interpretation is a terminal water ligand to Mn(III). These investigations thus allow the complete assignment of the protonation state of the Mn(III)/Fe(III) intermediate in *Ct* RNR: a μ-oxo/μ-hydroxo core with a terminal water ligand to Mn(III). This structural elucidation sheds additional light on proton-coupling to the RT that is central to class I RNR catalysis in *C. trachomatis*, the role of protonation in modulating the reactivity of metallo-cofactors, and the reactivity of heterobimetallic Mn/Fe active sites in nature.

## Materials and Methods

### Sample Preparation

Wild-type *Ct* RNR β (UniProt O84835) and *Ct* RNR Δ(1–248)α (UniProt O84834) were heterologously produced in *E. coli* using pET28a vectors and purified to homogeneity via nickel affinity chromatography according to methods previously described.^25,73^ As in prior studies, the N-terminal 248 residues of the α subunit were truncated to improve expression and solubility. Proteins were rendered metal-free by dialysis of Δ(1–248)α against buffer containing EDTA and by treatment of β with the iron chelator, ferrozine, which was subsequently removed using a Sephadex G-50 column.^67,73^ Reconstitution of metal-free *Ct* β with Mn and Fe was performed in a 1:1 ratio according to the methods previously reported.^67,73^

Removal of O_2_ from preparations of *Ct* α and β was performed as described previously.^91^ Where applicable, exchange of protein into ^2^H_2_O buffer (100 mM sodium HEPES, pD 8.0) was accomplished by 10-fold dilution of concentrated protein solution with ^2^H_2_O buffer and subsequent reconcentration in an Amicon ultrafiltration cell; this process was performed three times. The Mn(III)/Fe(III) state was prepared by initiation of the reaction in the presence of hydroxyurea, also as previously described.^84^ EPR samples were prepared, transferred to quartz tubes for X-band (4.0 mm O.D., 3.0 mm I.D.) and Q-band (2.8 mm O.D., 1.8 mm I.D.) measurements, and subsequently frozen in liquid N_2_.

### EPR Measurements

X-band, CW EPR measurements were performed on a Freiburg Instruments Magnettech MS-5000X spectrometer integrated with a Bruker ER 4112-HV variable temperature helium flow cryostat via a custom framework. The temperature was controlled by a LakeShore 335 Cryogenic Controller. We estimate ±2 K error margins in the measured temperature in these experiments, with the exception of the temperature dependence of power saturation. In these latter experiments, the temperature at the position of the sample was calibrated by an external temperature sensor (LakeShore Cernox CX 1050) placed in a quartz EPR tube before and after the execution of the experiment. To minimize the temperature gradient across the sample, these measurements were performed at high helium flow (∼1.5 L/h). Under these conditions, we estimate the temperature measurement error margins to be ±0.25 K.

Q-band measurements were performed on a Bruker Elexsys E580 X-band spectrometer equipped with a SuperX-FT microwave bridge in combination with an Oxford CF935 helium flow cryostat. Q-band frequencies were acquired using a home-built intermediate-frequency extension of the SuperX-FT X-band bridge equipped with a Millitech 5 W pulse power amplifier. Measurements were conducted on a home-built TE011 resonator utilizing the open resonator concept developed by Annino et al.^92^ and mechanical construction of the probe-head similar to that presented by Reijerse et al.^93^ The resonator contains ENDOR coils comprised of four silver posts in a Helmholtz post arrangement. This setup allows *t*_π/2_ = 12–16 ns at maximum input power with a spectrometer dead time (including the resonator ring time) of 100–120 ns. Radio frequency radiation was controlled by a Bruker DICE RF synthesizer and amplified by a BT01000-AlphaSA TOMCO 1 kW amplifier. Unless otherwise noted, π/2 pulses were 12 ns. Data acquisition and control of experimental parameters were performed using Bruker XEPR software.

The following pulse sequences were employed:

FID-detected EPR: [π/2]-*t*_deadtime_-detection.

HYSCORE: [π/2]-τ-[π/2]-*T*_1_-[π]-*T*_2_-[π/2]-τ-detection.

Mims ENDOR (refocused):^89^ [π/2]-τ-[π/2]-*t*_d1_-[π_RF_]-*t*_d2_-[π/2]-*t*_d3_-[π]-(*t*_d3_-τ)-detection.

### EPR Analysis

Data processing and spectral simulations were performed using Kazan Viewer, a home-written suite of utilities in MATLAB (MATLAB r2022a, The Mathworks Inc.). One-dimensional EPR simulations were performed using the “pepper” and “esfit” utilities from the EasySpin software package.^94^ HYSCORE and ENDOR data were analyzed by simultaneous frequency-domain simulation of all field-dependent spectra until a satisfactory solution was achieved. Euler angles are reported according to the “*y*” convention; if unspecified, these angles are [0, 0, 0]°. For additional details on EPR fitting and simulation, see Supporting Information.

## Supporting Information Available

Additional methodological details regarding fitting of CW EPR line broadening, power saturation experiments, and simulation of ^1^H-ENDOR spectra; additional CW EPR, HYSCORE, and ENDOR figures; table of fitting parameters (PDF)
