# Supplementary material for: Structural Elucidation of the Reduced Mn(III)/Fe(III) Intermediate of the Radical-Initiating Metallocofactor in Chlamydia trachomatis Ribonucleotide Reductase
Source: Biochemistry. 2025 Feb 17;64(5):1157–67. doi: 10.1021/acs.biochem.4c00692 (PMC11883743; doi:10.1021/acs.biochem.4c00692)
Supplement: Supplementary file 1 — bi4c00692_si_001.pdf [file bi4c00692_si_001.pdf]

Supporting Information for:

Structural elucidation of the reduced Mn(III)/Fe(III) intermediate of the radical-initiating metallocofactor in *Chlamydia trachomatis* ribonucleotide reductase

Ryan J. Martinie,[a]* Jovan Livada,[b] Nyaari Kothiya,[a] J. Martin Bollinger, Jr.,[b,c,ɸ] Carsten Krebs,[b,c] and Alexey Silakov[b]*
[a]Department of Chemistry, Hamilton College, Clinton, NY USA 13323.
[b]Department of Chemistry, The Pennsylvania State University, University Park, PA USA 16802.
[c]Department of Biochemistry and Molecular Biology, The Pennsylvania State University, University Park, PA USA 16802.
[ɸ] Contributing author for special issue honoring Christopher T. Walsh
*To whom correspondence should be addressed: rmartini@hamilton.edu, aus40@psu.edu

Table of Contents

Supplementary Materials and Methods……………………………………………………… …………. S3
Supporting Figures………………………………………………………………………………… S6
Supporting Tables…………………………………………………………………………………S12
Literature Cited……………………………………………………………………………………S13

Materials and Methods

Fitting line broadening of X-band continuous wave EPR spectra in H_2O and D_2O

In order to evaluate whether the strongly coupled signal observed in the HYSCORE spectra might arise from a single strongly coupled deuteron or from two deuterons with similar coupling parameters, we quantitatively evaluated the line broadening in X-band CW EPR spectra for samples prepared in H_2O and D_2O. To do so, the spectra were fit using a least-squares minimization approach using the "esfit" function of the EasySpin software package.[1]

The overall approach was to fit the D_2O spectrum, including line broadening parameters, then use these same parameters to fit the H_2O spectrum, allowing only the HStrain (residual, anisotropic line broadening parameter that is differentiated for each of the principle components of the *g* tensor) to float. Using the esfit function of EasySpin, the following best fit was obtained for the D_2O spectrum: g = [2.0263, 2.0158, 2.01085], A_{55Mn} = [267.1, 394.6, 316.6], isotropic linewidth = 1.64 mT (FWHM), HStrain (anisotropic residual line width, FWHM) = [4.8, 19.4, 15.4] MHz (Table S1). It should be noted that these parameters have minor deviations compared to those reported in the main text, which resulted from global simulation of the X- and Q-band one-dimensional spectra; rather than use these global parameters unaltered, the X-band spectra were fit to obtain the ideal simulation for the samples being compared. In order to ensure that the presence of a minor radical contaminant did not interfere with the fitting, this contaminant was also fit with the following parameters: g_{iso} = 2.0006, isotropic linewidth = 0.3 mT (FWHM), weight = 0.0001 [relative to Mn(III)/Fe(III)]. The same parameters were used as a starting point in fitting the H_2O spectrum, and the radical contaminant was fit, refining to: g_{iso} = 2.002, isotropic linewidth = 0.3 mT (FWHM), weight = 0.0001. With the contaminant parameters set, the HStrain was then allowed to float for the H_2O spectrum, with a best fit of [20.7,30.6, 33.6] MHz. This entire analysis (both D_2O and H_2O fitting) was performed multiple times from varying starting parameters, and yielded consistent changes in HStrain (± 15%). Example best fit parameters are compiled in Table S1.

The difference in HStrain between the two samples is attributable to the change in hyperfine couplings when exchangeable protons are replaced with deuterium nuclei in the D_2O sample. For simplicity, we will begin with the assumption that all change in linewidth is due exclusively to the presence of the hydron(s) that exhibit the large coupling observed in HYSCORE experiments, neglecting any weaker couplings. Since deuterium has a 6.51-fold weaker magnetic moment compared to a proton, but has double the overall splitting for the same hyperfine coupling due to its greater nuclear spin (I = 1 vs 1/2), the increase in linewidth will be ΔHStrain = n*4.51*$|A_{2H}|$, where A_{2H} is the hyperfine coupling to the deuterium and n is the integer number of deuterium nuclei exhibiting this coupling. Given that the strong coupling observed in ^{2}H-HYSCORE can be simulated with $|A_{2H}|$ = [3, 4, 4] MHz and ΔHStrain = [16, 12, 19] MHz, therefore [16, 12, 19] = n*[14, 18, 18]. Clearly, the closest integer value of n is 1.

If n = 2, then $|A_{2H}|$ would be expected to be ~[1.8, 1.3, 2.1] MHz based on the CW line broadening we observe. Since other, smaller hyperfine couplings also contribute to the change in linewidth, this would represent an upper bound for the large hyperfine coupling. Therefore for n = 2, $|A_{2H}|$ < [2, 1, 2] MHz; this magnitude of $|A_{2H}|$ is clearly incompatible with the ^{2}H-HYSCORE results reported here (Figure 3, main text). Overall, this analysis strongly suggests that the large coupling observed in ^{2}H-HYSCORE experiments arises from a single hydron in the Mn(III)/Fe(III) state.

Fitting of temperature-dependent saturation behavior

Power saturation of the double integral of the CW EPR signal (DI) follows the following law:

$$DI = \frac{\sqrt{P}A}{\left[1+\left(\frac{P}{P_{0.5}}\right)\right]^{b/2}} \quad (1)$$

where A is an instrument-dependent constant that also includes the spin concentration, b is the inhomogeneous line broadening parameter and ranges from a value of 1 (fully homogeneous) to 3 (highly inhomogeneous), and $P_{0.5}$ parameter is proportional to the temperature-dependent longitudinal electron spin relaxation rate. Due to the presence of minor contaminants in the region of g ~ 2, we approximated DI as the double integral of the two lowest-field packets of the CW EPR spectrum. In our hands, best fit was obtained if $DI(\sqrt{P})$ and $\frac{DI(\sqrt{P})}{\sqrt{P}}$ were fit simultaneously with equal weight. The homogeneity broadening parameter b was set to 1 in all fits as it gave the most faithful simulation of the experimental data.

The $P_{0.5}(T^{-1})$ dependence plotted on a $\log_{10}(P_{0.5})$ scale (see Figure 4, main text) shows a linear dependence indicative of an Orbach relaxation process that depends on the energetic proximity of the first excited spin state (S = 3/2 in this case). To obtain the energy gap between the ground and first excited electron spin state, the following formula was used to fit the experimental data accounting for the Orbach relaxation process:

$$P_{0.5}(T) = \frac{A}{e^{\frac{\Delta}{kT}} - 1} \quad (2)$$

where A is a system-dependent fitting parameter and k is the Boltzmann constant. This fit yielded A = 9.73 ± 0.7 W and Δ = 82 ± 4 cm^{-1}. As has been described before, zero-field splitting terms from Mn(III) and Fe(III) ions can be neglected. Therefore, using the $\hat{H}_{exch} = J\mathbf{S}_{Fe}\cdot\mathbf{S}_{Mn}$ formalism for the Heisenberg exchange interaction part of the spin Hamiltonian, Δ corresponds to 3/2J, yielding $|J| = 54 \pm 3$ cm^{-1} in our case. All values of J both here and in the main text are reported based on the $\hat{H} = J\mathbf{S}_1\cdot\mathbf{S}_2$ convention; literature values reported according to the $\hat{H} = 2J\mathbf{S}_1\cdot\mathbf{S}_2$ convention have been adjusted accordingly.

Attempts to simulate ^{1}H-ENDOR spectra with a single set of hyperfine coupling parameters

First, ^{1}H-ENDOR spectra for Mn(III)/Fe(III) prepared in D_2O were subtracted from those prepared in H_2O (thus yielding ^{1}H-ENDOR spectra specific to protons in exchangeable positions adjacent to Mn(III)/Fe(III), Figure S5). Because the spectra were recorded on different samples with modestly different spectral intensities, the D_2O spectra were scaled so as to match, but never exceed, the intensity of the H_2O spectra (a signal of greater intensity *at any point in the spectra* would indicate the presence of a ^{1}H coupling that was more intense in D_2O than in H_2O, which is chemically implausible).

Upon examination of the subtracted spectra, the most striking features are the persistent sharp peaks with a splitting of ~4 MHz, present in spectra **1-4** from 1169.8-1177.2 mT (Figure 5, Figure S5 and S6). In the context of dimetal sites, protons with a hyperfine coupling of this relatively small magnitude are expected to be coupled via a dipolar mechanism and therefore exhibit approximately axial coupling parameters. Simulations with A_{1H} = [4.1, 4.1, -8.2] MHz nicely reproduce the sharp features (Figure S6B-C). Moreover, the "feet" of the Pake patterns overlay relatively well with some of the shoulders at larger splittings (~8 MHz). Crucially, however, spectrum **5** (1179.6 mT) exhibits well-defined "cliff-shaped" features with a splitting of ~6 MHz. Regardless of the manipulation of the Euler angles, a single, axial dipolar coupling either produces features for this spectrum that are far too narrow (~4 MHz, Figure S6B) or are too wide/broad (~8 MHz, Figure S6C).

Protons that are dipolar coupled to a dimetal center can exhibit somewhat rhombic hyperfine coupling parameters, due to the overall coupling being a sum of the dipolar hyperfine couplings to each of the two metal centers, weighted according to their spin projection factors.[2,3] This rhombicity increases when the proton is located relatively centrally (i.e. both metals are coupled with significant magnitude, and one or the other does not dominate) and the coupling is relatively strong. To explore this possibility, we also attempted to simulate the spectra with parameters that are not strictly axial. Simulating the ENDOR spectra with A_{1H} = [4.1, 4.1, -6.0] MHz can reproduce both the sharp features at ~4 MHz splitting in spectra **1-4** and the ~6 MHz "cliffs" in spectrum **5**, but doing so results in loss of the shoulders in spectra **1-4** (1169.8-1174.7 mT, Figure S6D). In short, a single set of hyperfine parameters is inadequate to simulate the varying features of the experimental spectra.

By contrast, the two-proton simulation reported in the main text reproduces all the main features of the experimental spectra, including the sharp features at ~4 MHz in spectra from **1-4**, the "cliffs" at ~6 MHz in spectrum **5**, the broad shoulders in spectra **1** & **2**, and the double shoulders in spectra **3** & **4** (note particularly the features at ~46 and ~47 MHz in spectrum **3** and those at ~53 and ~54 MHz in spectrum **4**).

Finally, we note also the presence of at least one non-exchangeable coupling (Figure S5), with an overall breadth of ~7 MHz. We attribute this signal to one or more non-exchangeable protons on the histidine ligands to the metallocofactor.

Supporting Figures

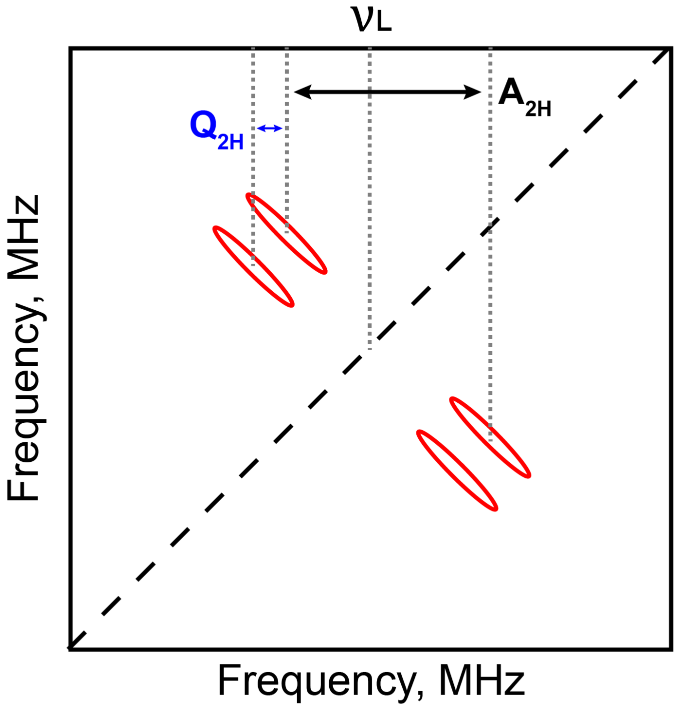

Figure S1. Cartoon representation of an example set of ^{2}H-HYSCORE crosspeak positions in the weak hyperfine coupling regime. A hypothetical HYSCORE signal consisting of four peaks (red) centered on the Larmor frequency (ν_L) exhibits splitting along the antidiagonal due to hyperfine coupling (A_{2H}) as well as the diagonal due to quadrupolar coupling (Q_{2H}).

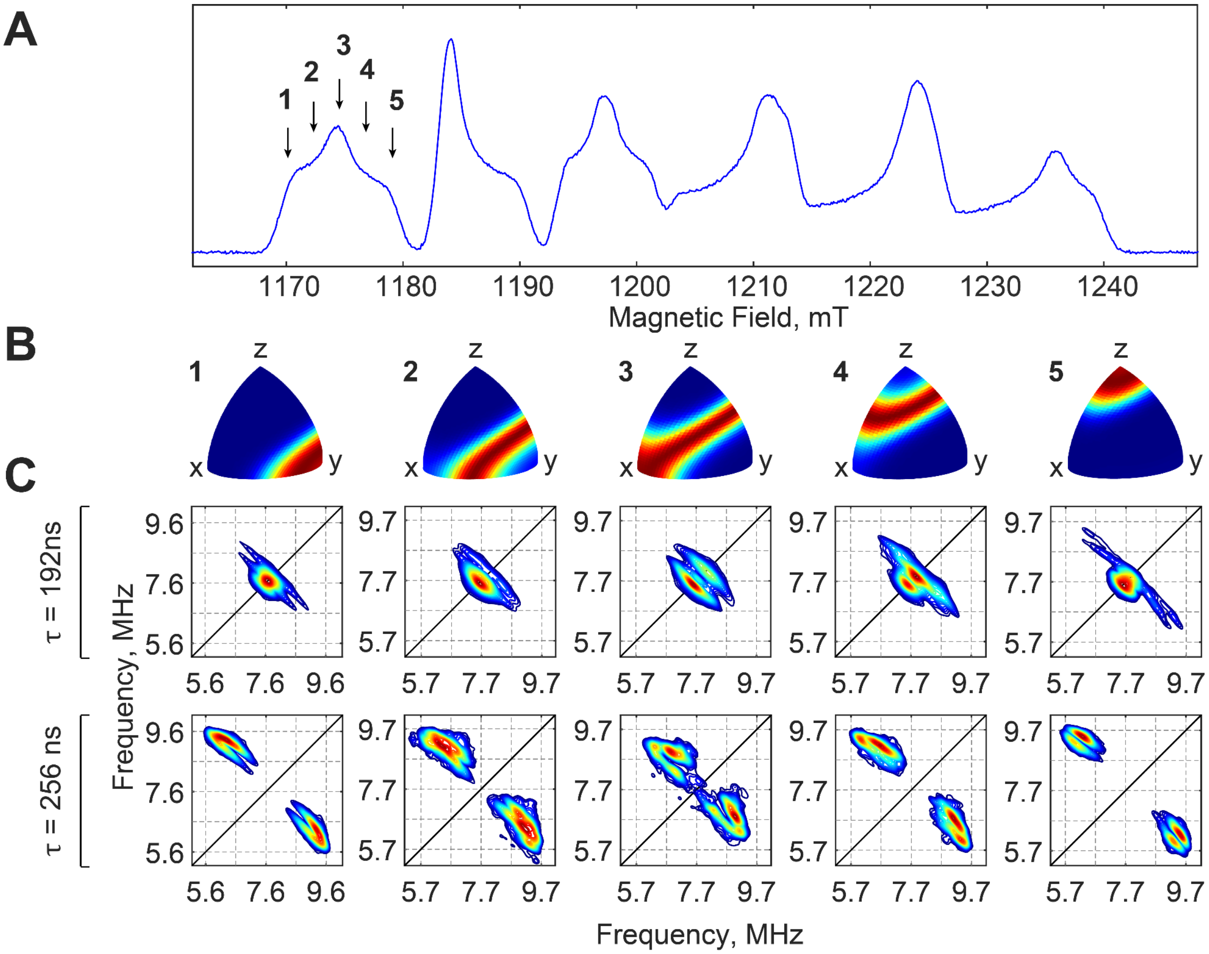

Figure S2. Orientation-selective, Q-band ^{2}H-HYSCORE characterization of the Mn(III)/Fe(III) state in *Ct* RNR prepared in ^2H_2O. **A**) EPR spectrum indicating the magnetic field positions (arrows, **1-5**) at which HYSCORE spectra were collected. Spectrum was acquired by monitoring the intensity of a free-induction-decay (FID) as a function of the magnetic field. **B**) Calculated orientation selectivity maps (**1-5**) resulting from HYSCORE acquisition at the magnetic field positions shown in **A**. Color coding: red, greatest excitation; blue, no excitation. **C**) Orientation-selective ^{2}H-HYSCORE spectra acquired at (from left to right): 1169.9 (**1**), 1172.0 (**2**), 1174.2 (**3**), 1176.7 (**4**), and 1179.1 (**5**) mT, microwave frequency 33.99 GHz, and τ = 192 ns (top row) or τ = 256 ns (bottom row). Minimum contour levels are set at 25% of the spectral maximum.

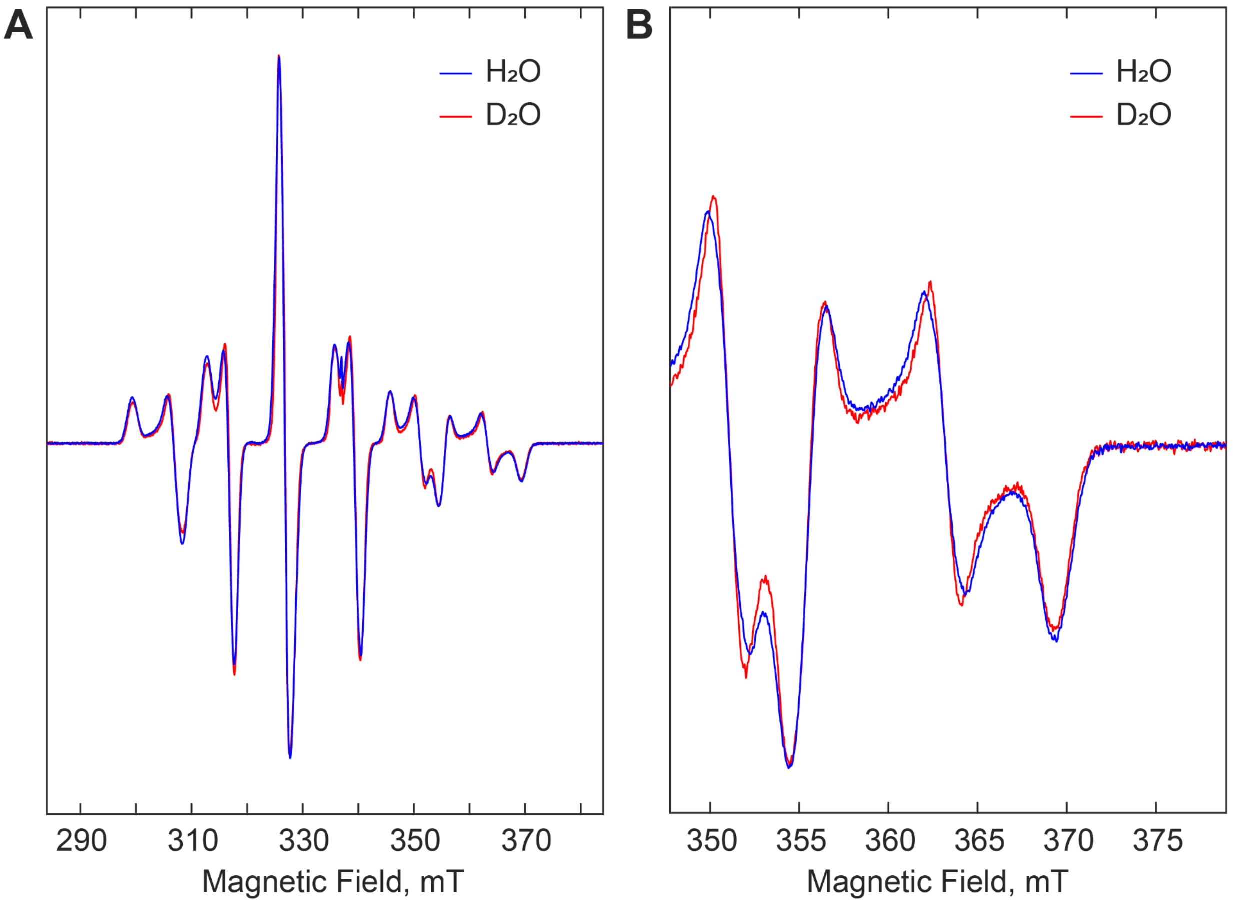

Figure S3. Comparison of continuous-wave EPR spectra for samples of *Ct* RNR Mn(III)/Fe(III) prepared in H_2O (blue) and D_2O (red). These samples yield nearly identical spectra (**A**), but broadening is apparent in the H_2O spectrum upon careful inspection of lineshapes (**B**). Spectra were acquired at a microwave frequency of 9.435 GHz, microwave power of 100 µW, modulation amplitude 0.15 mT, and a temperature of 15 K.

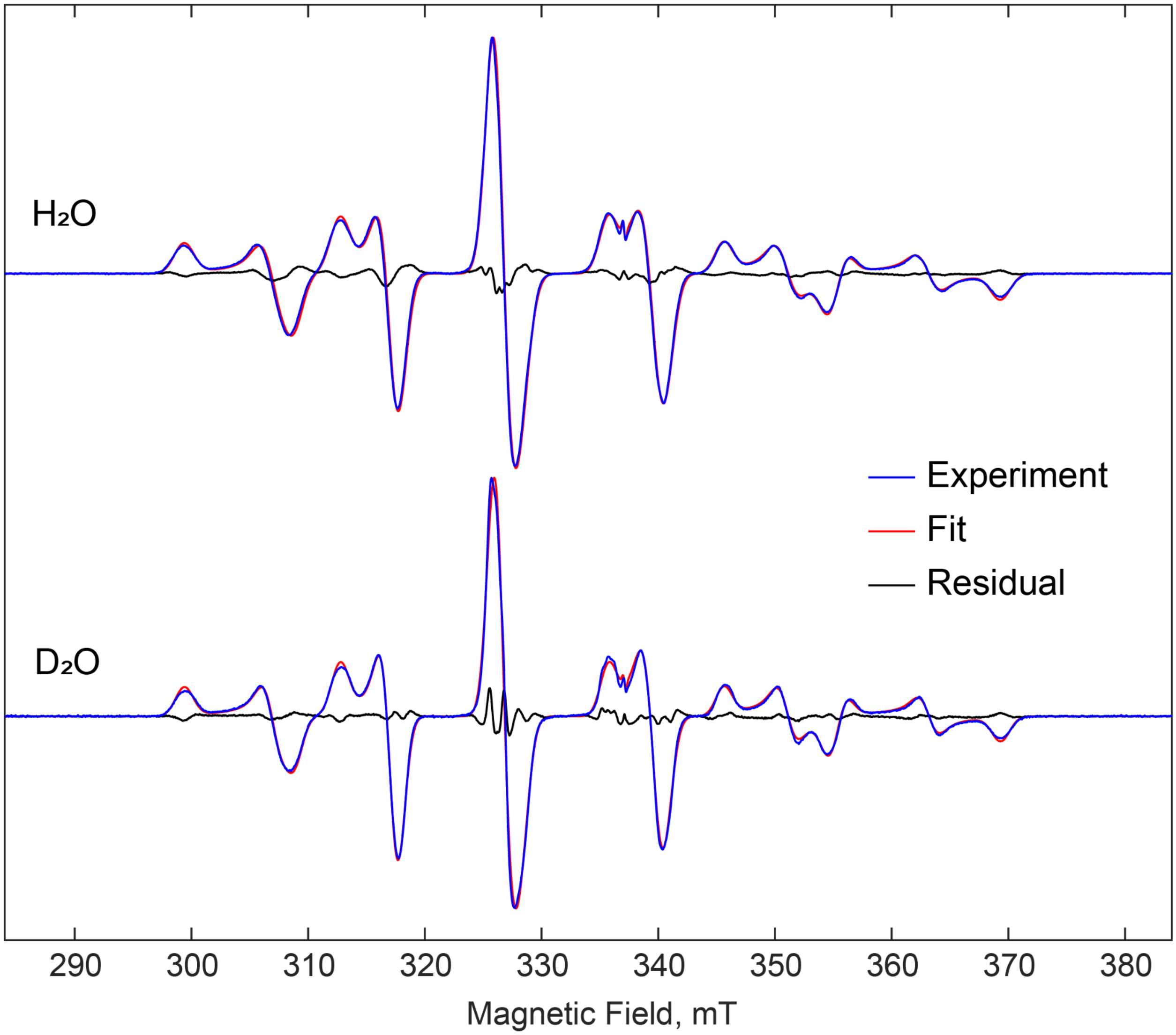

Figure S4. Comparison of X-band CW EPR spectra of *Ct* RNR Mn(III)/Fe(III) (blue) to fits generated using the esfit function of EasySpin (red), as well as residual (black). Comparisons are included for samples prepared in H_2O (above) and D_2O (below). Parameters of the simulations are reported in Table S1. Experimental parameters as in Figure S3.

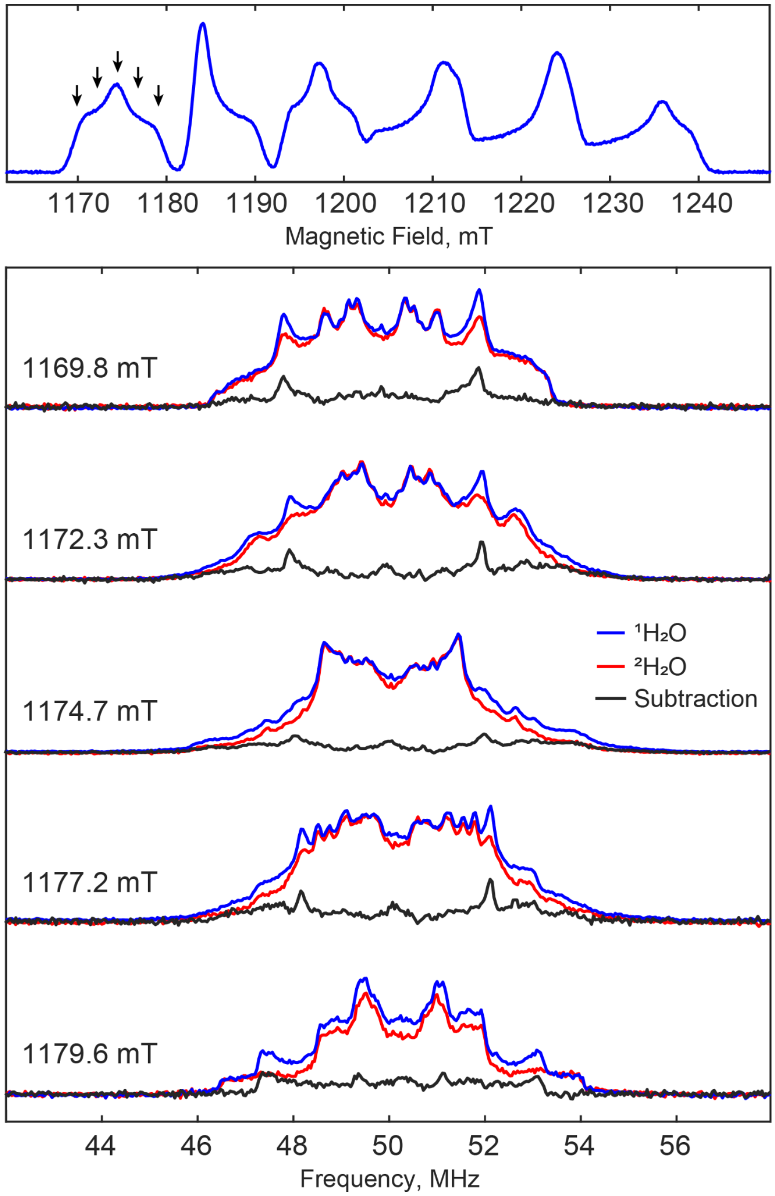

Figure S5. Comparison of magnetic-field-dependent, Q-band Mims ENDOR spectra collected on Mn(III)/Fe(III) samples prepared in $^{1}H_2O$ (blue) and $^{2}H_2O$ (red); subtractions of the $^{2}H_2O$ from the $^{1}H_2O$ spectra are also shown (black). Magnetic field positions of the ENDOR spectra are indicated by arrows on the EPR absorption spectrum (top). Spectra were collected with a microwave frequency of 33.991 GHz, 14 K, τ = 76 ns, 12 ns $\pi/2$ pulse, 12 μs RF pulse, and a shot repetition time of 1500 μs.

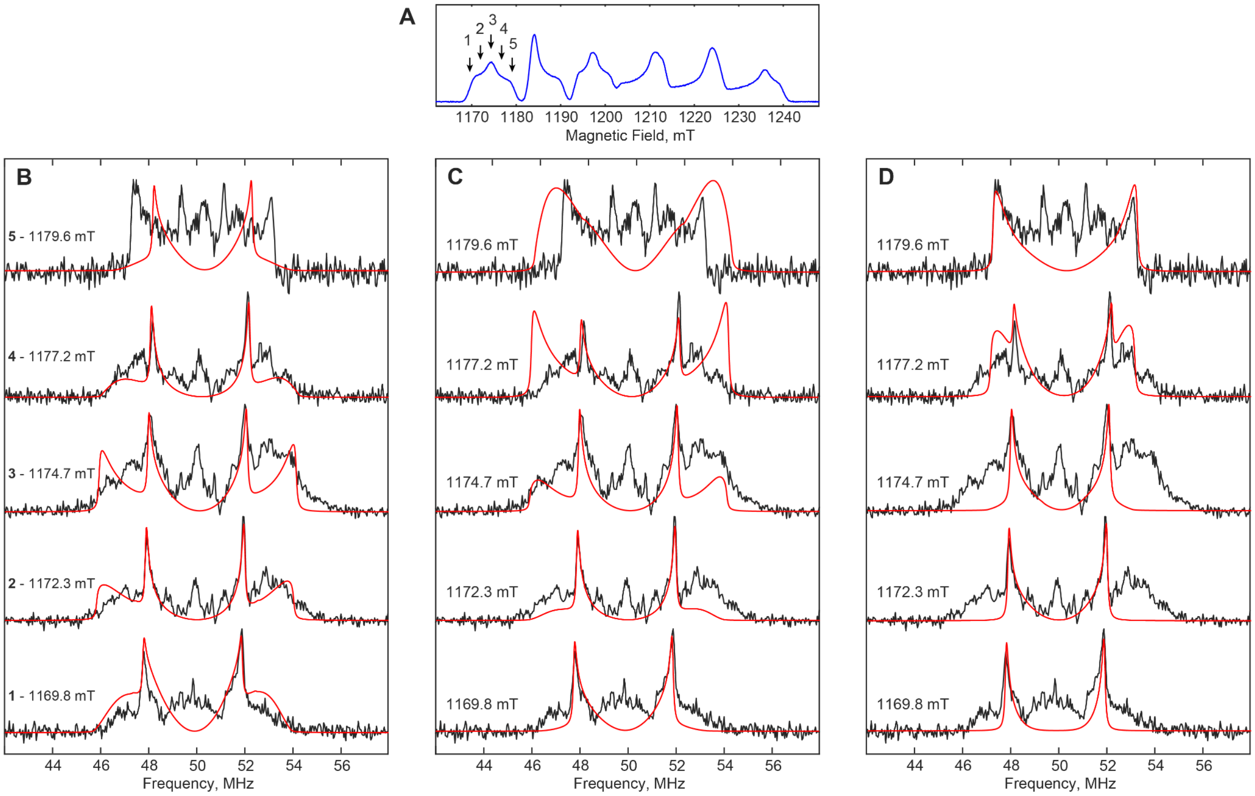

Figure S6. Simulations (red) of Q-band, ^{1}H-ENDOR spectra of the Mn(III)/Fe(III) state, assuming a single interacting ^{1}H nucleus and highlighting the inadequacy of such models to reproduce the experimental data (black). **A**) One-dimensional EPR spectrum indicating the magnetic field positions (arrows) at which ENDOR spectra were acquired. Spectrum was acquired by monitoring the intensity of a free-induction-decay (FID) as a function of the magnetic field. **B**) Magnetic-field-dependent refocused Mims ENDOR spectra (black) and simulation (red). Experimental data were acquired by subtracting the spectrum of a sample prepared in $^{2}H_2O$ from that of a sample prepared in $^{1}H_2O$, measured under identical conditions (see Figure S5). Central features (~50 MHz) arise from weak hyperfine couplings attributable to matrix protons. Experimental parameters as in Figure S5. Simulation according to the following parameters: A_{1H} = [4.1, 4.1, -8.2] MHz with Euler angles [0, 60, 45]°. **C**) Experimental spectra identical to those in B, but with the following simulation parameters: A_{1H} = [4.1, 4.1, -8.2] MHz with Euler angles [0, 40, 45]°. **D**) Experimental spectra identical to those in B & C, but with the following simulation parameters: A_{1H} = [4.1, 4.1, -6] MHz with Euler angles [0, 30, 90]°.

Supporting Tables

Table S1. Parameters from a representative best fit of CW EPR spectra at X-band for Mn(III)/Fe(III) samples prepared in H_2O and D_2O.

	**D_2O**	**H_2O**
g(1)	2.0263	2.0263
g(2)	2.0158	2.0158
g(3)	2.01085	2.01085
$A_{55Mn}(1)$	267.1	267.1
$A_{55Mn}(2)$	394.6	394.6
$A_{55Mn}(3)$	316.6	316.6
lw	1.64	1.64
Hstrain(1)	4.8	20.7
Hstrain(2)	19.4	30.6
Hstrain(3)	15.4	33.6

Literature Cited

(1) Stoll, S.; Schweiger, A. EasySpin, a Comprehensive Software Package for Spectral Simulation and Analysis in EPR. *J. Magn. Reson.* **2006**, *178* (1), 42–55. DOI:10.1016/j.jmr.2005.08.013.

(2) Bencini, A.; Gatteschi, D. *EPR of Exchange Coupled Systems*; Springer-Verlag: Berlin, 1990.

(3) Martinie, R. J.; Blaesi, E. J.; Krebs, C.; Bollinger, J. M., Jr.; Silakov, A.; Pollock, C. J. Evidence for a Di-μ-Oxo Diamond Core in the Mn(IV)/Fe(IV) Activation Intermediate of Ribonucleotide Reductase from *Chlamydia trachomatis*. *J. Am. Chem. Soc.* **2017**, *139* (5), 1950–1957. DOI:10.1021/jacs.6b11563.

(4) Dassama, L. M. K.; Krebs, C.; Bollinger, J. M., Jr.; Rosenzweig, A. C.; Boal, A. K. Structural Basis for Assembly of the MnIV/FeIII Cofactor in the Class Ic Ribonucleotide Reductase from *Chlamydia trachomatis*. *Biochemistry* **2013**, *52* (37), 6424–6436. DOI:10.1021/bi400819x.
